# The Building of Atlanta

**Published:** 1899-12

**Authors:** Bernard Wolff

**Affiliations:** Atlanta, Ga.


ATLANTA

### Journal-Record of Medicine.

Successor to Atlanta Medical and Surgical Journal, Established 1855, and Southern Medical Record, Established 1870.

VOL. I. DECEMBER, 1899. No. 9.

EDITORS { BERNARD WOLFF, M.D., DUNBAR ROY, M.D. M. B. HUTCHINS, M.D., BUSINESS MANAGER.

PUBLISHED MONTHLY. OFFICE 305 AND 306 FITTEN BUILDING.

### *ORIGINAL COMMUNICATIONS.*

### THE BUILDING OF ATLANTA.

Behold the stumps of innumerable chimneys,
Piles of brick and charred timbers overgrown with dockweed and dog-fennel!
Dewberry vines, like connective tissue, bind together
Warped metal, twisted pipes, ruined utensils of domesticity.
Heterogeneous homogeneity; the Apotheosis of Desolation, the blight of the scorching touch of Sherman's devastating legions.

The long curling crack of the teamster's black snake
Urges the toiling horses to drag the broad-tired dray
Through the red-gashed road, hub-deep in tenacious mud.
The tense muscular contraction throws the skin of the horses' rumps into folds and ridges
Until the indolent ulcers on their backs fissure and bleed,
Labor with pain.

The panorama slowly shifts.
Out of the desert wastes of the fire-fiend's dominion
Comes the incisive ring of hammers;

Houses spring from blackened, sunken cicatrices,
Fungoid, exuberant in rapid growth.
Work, energy, everywhere; a *furor operandi.*
Streets spread out like radii from the diaphanous home of the spider.
Progress swift, inexorable.
Sweetness from bitter—freshness from filth.
Despite the skulking scalawag and the iron beak and cruel talons of the carpet-bag condor—
The political vulture, bald, disgusting,
His crimson wattles flecked with gouts of blood from his torn, eviscerated victims;
Despite the alien hordes of the army of post-bellum occupation,
Which swollen and tight with the juices of the native people,
Were smashed between progress and patriotism
Musquito-like, and the unripe ova
Left to shrink and desiccate on a loyal soil
That would not absorb nor incubate them.

A Christ-like span of years divides the differentiated from the inchoate,
Years in numerical arrangement of time, æons of change,
Change whose emblems are iron and brick and mortar,
Not cobwebs, flittermice, mildew and rusted hinges.
The lamp of Aladdin, the wand of Circe, the great fane on the sometime threshing floor of Ornan, the Jebusite.
Gone is the laggard step and the opulent sloth of yore.
Gone before the solvent breath of the Zeit-Geist—the spirit of constructive mutation,
That which urges the germinal principle of life into blossom;
That disposes in order the embryonal conglomerate,
What time the quickened womb casts forth the finished product.
That abides in the wheeling stars and the marching suns;
That rears the giant oak and sharpens the gnawing teeth of the squirrel;
That directs the whirring flight of the insect;
That grooves the recurved fang of the crotalus;
That gives irridescence to the chromatic vortices of the peacock;
That focalizes discontent till the snarl of the tike of discord
Is drowned in the growl of the thick-lipped mastiff of war.
Design, the purpose of God; constructive change, the tool of innumerable handles
With which the Master Craftsman does his perfect work.
So the eternal principle of progress working in the strenuous hand of man and the will of God
Upbuilt the city, and so each burly structure
Becomes an emblem of faith in the soil and a confident hope of the future.

Bernard Wolff.

